# The mechanism of olfactory organ ventilation in *Periophthalmus barbarus* (Gobiidae, Oxudercinae)

**DOI:** 10.1007/s00435-012-0167-y

**Published:** 2012-07-18

**Authors:** Michał Kuciel

**Affiliations:** Department of Comparative Anatomy, Institute of Zoology, Jagiellonian University, ul. Gronostajowa 9, 30-387 Kraków, Poland

**Keywords:** Olfactory organ, Olfaction, Mudskipper, Oxudercinae, Functional morphology

## Abstract

*Periophthalmus barbarus* Linnaeus, 1766 has many adaptations for amphibious life as a consequence of tidal zone occupation. One of them is the ability to keep a little amount of water and air in mouth while on land or in hypoxic water, correlated with closing a gill lid for gas exchange improvement. It causes that mechanisms of olfactory organ ventilation described in other species of actinopterygians (compression of accessory nasal sac(s) by the skull and jaw elements while mouth and gill lid moving) are not in operation. There is a specific mechanism of olfactory organ ventilation independent on jaw and skull elements movements. Compression of accessory nasal sacs is possible by *a0* contraction and it is a movement effect on bones combined by ligaments. This process can be observed on *P. barbarus* as lifting the rostral part of the head.

**Electronic supplementary material:**

The online version of this article (doi:10.1007/s00435-012-0167-y) contains supplementary material, which is available to authorized users.

## Introduction

The structure and/or functioning of the olfactory organ have been described in the basally branching gnathostome vertebrates, the Actinopterygii (Burne 1909; Chabanaud 1927; Teichmann 1954; Kapoor and Ojha 1972, 1973; Bashor et al. 1974; Zeiske 1974; Døving et al. 1977; Jakubowski and Kunysz 1979; Melinkat and Zeiske 1979; Zeiske et al. 1979; Theisen 1982; Yamamoto 1982; Applebaum and Schemmel 1983; Kux et al. 1988; Sinha and Sinha 1990; Webb 1993; Eastman and Lanoo 2001; Belanger et al. 2003; Chakrabarti and Hazra Choudhry 2006; Kumari 2008; Kuciel et al. 2011) and Elasmobranchii (Burne 1909; Teichmann 1954; Døving et al. 1977). Many of these species have developed a mechanism involving the passive movement of water through the nose while fish stay in water flow or as a result of ciliary beating (isomates). In other species, water flow through the olfactory organ is regulated actively by the compression of accessory nasal sacs that penetrate between the bones of the skull and jaw apparatus (cyclosmates) (Døving et al. 1977).

Olfactory organs of most actinopterygians consist of olfactory rosettes located in olfactory chambers. The olfactory chambers are always on the dorsal side of the head between the eyes and the end of the snout, while the olfactory chambers of elasmobranch species, with the exception of the Chlamydoselachidae, are present on the underside of the head, before the mouth. A more advanced structure of the olfactory apparatus, in which the olfactory chamber is connected to a cavity, was described in Dipnoi, an extinct Crossopterygian, and several representatives of *Gymnodraco*, *Astroscopus* and *Ophichthidae* (Jakubowski 1975; Andriaschev et al. 1989). Water with dissolved particles is tested first by the epithelial olfaction receptor within this cavity and later by the epithelial taste receptor.

Oxudercinae amphibious species, like *P. koelreuteri*, spend up to 90 % of their life time on land (Gordon 1969). During this period, a small volume of moist air, and in some species, like *Periophthalmus chrysospilos* Bleeker, 1852, also a small volume of water (to improve gas exchange) are maintained in the mouth and gill cavity due to a tightly closed mouth and gill lid (Lee et al. 1987). This behavior was also observed while in hypoxic water. During this time, the gill lid and mouth do not move, excluding the possibility of compression of the chamber-like sacs during gill ventilation or mouth opening as reported in Parker (1910), Pipping (1926), Liermann (1933), Melinkat and Zeiske (1979), Nevitt (1991).

*Periophthalmus barbarus* is an amphibious species naturally occurring in the intertidal zone of West Africa. Recently, the unique structure of the olfactory organ in this species was described (Kuciel et al. 2011). It consists of a tube-like elongated canal transforming into a chamber-like sac, which is functionally analogous to the accessory nasal sac. The canal starts with a roundish anterior nostril on the verge of the upper lip and gradually widens transforming into a chamber-like sac. There is a posterior nostril in the chamber-like sac. The olfactory sensory epithelium is present only in the form of islets at the inner wall of the tube-like elongated canal.

The supply of water with ligands to the olfactory organ in *P. barbarus* is difficult or even impossible to maintain due to the small diameter of the channel (0.3 mm) (Kuciel et al. 2011), and the periodic cessation of compression of the chamber-like sacs as a result of movements of the mouth and gill lid. For this reason, there an independent mechanism for regulating sac compression by the aforementioned movement of the mouth and gill lid could be expected. In order to test this assumption, we studied the mechanism of olfactory organ ventilation in *P. barbarus*.

## Materials and methods

The study was conducted on 3 specimens of *Periophthalmus barbarus* Linnaeus, 1766 (Gobiidae, Oxudercinae) of total body length (TL) ranging from 7.6 to 13 cm. All specimens were bought in pet shop in good condition. They were kept in aqua-terraria, at a temperature of 28 °C, water salinity of 25 % and fed with insects and tubifex. Primarily observations on land and in water were made on live specimens and then anatomical study started.

Photographs showing contractions and elevation of the rostral part of the head while on land were made by a Panasonic Lumix DMC-FZ 50 digital camera.

Specimens were anesthetized with aqueous tricaine solution (0.1 % MS 222, Sigma Aldrich) and fixed in formalin (4 %). Two specimens were preserved in order to capture “movement” between the olfactory organ and its attendant ligaments and other structures associated with its functioning. Prior to dissection, two fixed specimens were first rinsed in distilled water and then dissected using a stereomicroscope magnifying glass MST-130.

To obtain a skeleton preparation, one fixed specimen was subjected to a 2 % aqueous potassium hydroxide solution. After 14 days, a few drops of alizarin red were added to color the bones and the preparation was transferred to glycerin. Photographs and drawings of the head were made from these preparations after removing soft tissues.

## Results

### Location of chamber-like sacs in relation to the surrounding structures

**Fig. 1:**
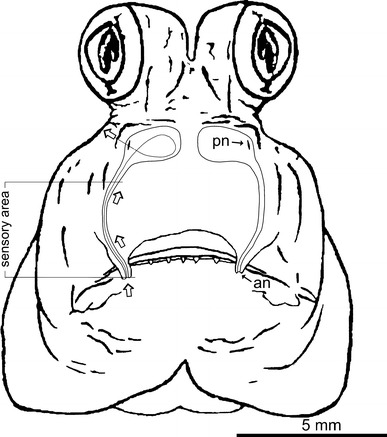
Schematic location of the olfactory organ in *P. barbarus*. *Arrows* and the *line* indicate the direction of water flow into the organ, *an* anterior nostril, *pn* posterior nostril

**Fig. 2:**
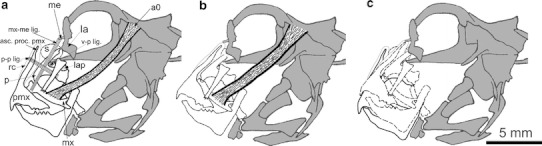
Diagram illustrating the organization (**a**) and movement (**b**, **c**) of the structures associated with the functioning of the olfactory organ. *S* chamber-like sac, *lap* levator arcus palatini, *a0* adductor mandibularis, *me* mesethmoideum, *la* lacrimal, *p* palatinum, *pmx* premaxillaria, *asc.proc.pmx.* ascending process of the premaxillary, *mx* maxillaria, *rc* rostral cartillage, *mx*-*me lig.* maxillaria–mesethmoideum ligament, *p*–*p lig.* ligament of two palatinum processes, *v*-*p lig.* vomer-palatinum ligament. In (**c**) *a0* removed for better view of bones movements (supplementary material 1)

**Fig. 3:**
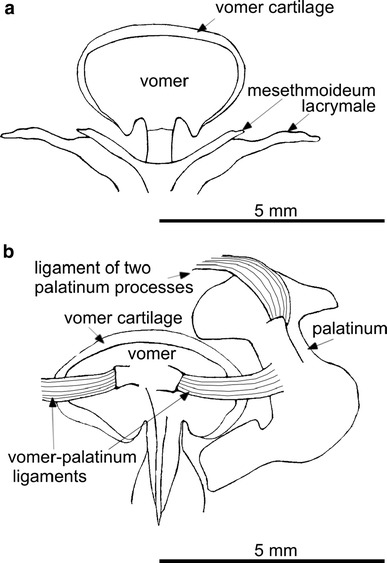
Dorsal (**a**) and ventral (**b**) part of the vomer (supplementary materials 2 and 3)

**Fig. 4:**
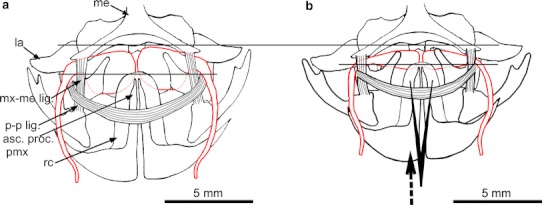
The elements involved in the regulation of the compression of the chamber-like sacs (**a**) and (**b)** the forced displacement of the ascending process of the premaxillary in a caudal direction (*arrow*) using tweezers to the first weak resistance. The distance between the lines illustrates the diminishing space occupied by the chamber-like sacs. *me* mesethmoideum, *la* lacrimal, *rc* rostral cartillage, *mx*-*my lig.* maxillaria–mesethmoideum ligament, *acs. proc.pmx.* ascending process of the premaxillary

The olfactory organ of *P. barbarus* is paired and symmetrically located in the rostral part of the head (Fig. 1). Each organ consists of a channel transforming into a chamber-like sac. Thin-walled, surrounded by connective tissue chamber-like sacs are located in the space between the bones and cartilage and are covered by skin (Fig. 4a, b). From the buccal side of the chamber-like sac, there is a V-shaped palatinum bone (Figs. 2a, 3b). A rounded vomer surrounded by cartilage is found on the ventral side (Fig. 3a, b), whereas lacrimal and mesethmoideum bones are located on the caudal side of the chamber-like sacs (Figs. 2a, 3a, 4a). In the fronto-medial part of the sacs, conical rostral cartilage is located at the spinous process of the premaxillary bones (ascending process of the premaxillary) (Figs. 2a, 4a). Between the vomer and palatinum, maxillaria and mesethmoideum and between processes of two palatinum there are, respectively, ligaments, vomer-palatinum ligaments (Figs. 2a, 3b), maxillaria–mesethmoid ligaments (paired) (Figs. 2a, 4a), and processes of two palatinum ligament (singular) (Fig. 4a) encircling the front of the ascending process of the premaxillary (Figs. 2a, 3b, 4a).

### Operation

Ventilation of the olfactory organ in *P. barbarus* actively involves *levator arcus palatini* (*lap*) () and *adductor mandibularis* (*a0*) muscles. The *a0* muscle on one side is attached to the skull and on the other to the maxillaria (Fig. 2a). The *lap* muscle is attached on one side to the palatinum and on the other to the ventral side of the vomer.

Contraction of the *a0* muscle moves both the embedded maxillaria and palatinum in a dorso-inner direction. Odd ligament (palatinum–palatinum) transfers forward an *a0* muscle contraction, causing the displacement of the ascending process of the premaxillary with rostral cartilage in the caudal direction (Figs. 2b, c, 4b).

**Fig. 5:**
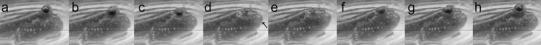
Lifting of the rostral part of the snout (*arrow*) arising from the movement of structures associated with the work of chamber-like sacs. *Line* the benchmark for the spots of the pectoral fins

Displacement of the upper part of the palatinum in the direction of centripetal and rostral cartilage (embedded in the ascending process of the premaxillary) toward the caudal side causes compression of the space occupied by the chamber-like sacs, and the rise of pressure, which pushes the water outside (Figs. 2a, b, c, 4b). Time-lapse images (Fig. 5a, b, c, d, e, f, g, h) shows the rostral part of the mouth rising, which is the result of movement of chamber-like sac related structures.

A new portion of water enters the olfactory organ by means of a negative pressure created in the organ after the disappearance of *a0* muscle contraction and the return of displaced elements to their initial positions (Figs. 2a, 4a). In addition, *lap* muscle contraction moves the lower part of the palatinum toward the vomer and the upper part of the palatinum (adjacent to the chamber-like sac) in the centrifugal direction increasing the space around the chamber-like sac. Uprising of underpressure is possible while a narrow, longitudinal slit-like posterior nostril is permanently closed what serves as a kind of one-way valve.

## Discussion

Our observations in vivo and examination of the supporting structures of the olfactory organ on anatomical preparations have pinpointed the probable scheme of water circulation in the olfactory organ. This process (1 cycle) runs in two stages: (1) suppression of chamber-like sacs by the palatinum and rostral cartilage of the ascending process of the premaxillary, resulting in pressure that pushes out the water present in the sacs through the posterior nostrils and (2) the moving parts of the cranial skeleton return to their original positions, producing a vacuum sucking a new portion of water into the tube-like elongated canal. These movements were observed both on land and in water and were often correlated with pulling eye to eye sockets. Observations of living mudskippers on land did not reveal leakage of water from the canal—olfacto-sensory part of the organ, which probably prevents its interior penetration by air.

The anatomical structure of fish olfactory organs has different adaptations and very much depends on the ecological niche occupied by the species in question. An example of a species with “simple” structure of the olfactory organ is the snook, *Belone belone*. The olfactory sensory epithelium is located on a fungiform structure embedded in a shallow groove, and the olfactory receptor cells are washed by water while swimming (Theisen et al. 1980). In most species, the functioning of the olfactory organ is more sophisticated. In species belonging to the group isosmates (Døving et al. 1977), the exchange of water in the organ is possible when specimen stay in water flow and may be improved by ciliary beating located within the olfactory sensory epithelium.

In contrast, cyclosmates (Døving et al. 1977) have accessory nasal sacs. If accessory nasal sacs develop in the vicinity of bones, then they are called ventromesial sacs (Kapoor and Ojha 1973) or lacrymal sacs (Burne 1909), and if at the median of the ethmoid, then they are termed ethmoidal sacs (Burne 1909) or dorsomesial sacs (Kapoor and Ojha 1973). In fish belonging to this group, regulation of water flow in the olfactory organ may occur partly through ciliary beating, but with the essential role of accessory nasal sacs. The number of accessory nasal sacs in a single organ varies from 1 to 2 depending on the species.

Volume changes of accessory nasal sacs are possible due to buccal muscle contractions causing jaw movement, or by pressure changes in the lymphatic system (van den Berghe 1929; Melinkat and Zeiske 1979). Nevitt (1991) described a reflex called “sniffing” which occurs very quickly and dynamically in two species of Pleuronectiformes [*Lepidopsetta bilineata* (Ayres, 1855), *Platichthys stellatus* (Pallas, 1787)], flushing the sensory olfactory epithelium.

Most of previously described species belonging to Perciformes possess two accessory nasal sacs, that is, a lateral lachrymal and medial ethmoidal sac (Burne 1909; Døving et al. 1977; Belanger et al. 2003; Sinha and Sinha 1990; Kapoor and Ojha 1972, 1973). One of few exceptions, with only one accessory nasal sac in between *Trachinus viper* and *Scomber scombrus* (Burne 1909), is *P. barbarus.*

The sacs perform only a mechanical function, forcing water to the tube-like elongated (sensory) part of the olfactory organ. In *P. barbarus*, the chamber-like sac determines the circulation of water in the channel and the proper functioning of the olfactory organ.

## Electronic supplementary material

Below is the link to the electronic supplementary material.

Alizarin stained *Periophthalmus barbarus* skull skeleton. In order to view skull bones, soft tissues were removed (EPS 4.90 mb)

Dorsal view of *Periophthalmus barbarus* alizarin stained vomer with mesethmoideum and lacrimale (EPS 3.59 mb)

Ventral view of *Periophthalmus barbarus* alizarin stained vomer with palatinum, vomer-palatinum ligament and fragment of process of two palatinums ligament (EPS 5.14 mb)
